# Paracoccidioidomycosis in Immunocompromised Patients: A Literature Review

**DOI:** 10.3390/jof5010002

**Published:** 2018-12-26

**Authors:** João N. de Almeida Jr., Paula M. Peçanha-Pietrobom, Arnaldo L. Colombo

**Affiliations:** 1 Central Laboratory Division, Hospital das Clínicas da Faculdade de Medicina da Universidade de São Paulo, CEP 05403-000 São Paulo, Brazil; jnaj99@gmail.com; 2 Department of Medicine, Division of Infectious Diseases, Escola Paulista de Medicina, Universidade Federal de São Paulo, CEP 04039-032 São Paulo, Brazil; paulampecanha@gmail.com

**Keywords:** paracoccidioidomycosis, HIV, cancer, lymphoma, kidney transplant, TNF inhibitors, literature review

## Abstract

*Paracoccidioidomycosis* (PCM) is an endemic mycosis found in Latin America that causes systemic disease mostly in immunocompetent hosts. A small percentage of PCM occurs in immunocompromised patients where low clinical suspicion of the infection, late diagnosis, and uncertainties about its management are factors that negatively impact their outcomes. We conducted a literature review searching reports on PCM associated to HIV, cancer, maligned hemopathies, solid organ transplantation, and immunotherapies, in order to check for peculiarities in terms of natural history and challenges in the clinical management of PCM in this population. HIV patients with PCM usually had low T CD4^+^ cell counts, pulmonary and lymph nodes involvement, and a poorer prognosis (≈50% mortality). Most of the patients with PCM and cancer had carcinoma of the respiratory tract. Among maligned hemopathies, PCM was more often related to lymphoma. In general, PCM prognosis in patients with malignant diseases was related to the cancer stage. PCM in transplant recipients was mostly associated with the late phase of kidney transplantation, with a high mortality rate (44%). Despite being uncommon, reactivation of latent PCM may take place in the setting of immunocompromised patients exhibiting clinical particularities and it carries higher mortality rates than normal hosts.

## 1. Introduction

Paracoccidioidomycosis (PCM) is a systemic endemic mycosis caused by *Paracoccidioides brasiliensis* and *Paracoccidioides lutzii*, exhibiting geographic distribution restricted to Latin America, mainly Brazil, Argentina, Colombia and Venezuela [1].

The real burden of the disease is still not defined but incidence rates ranging from 0.7–3 and 9–40 cases/100,000 inhabitants have been reported in endemic and hyperendemic areas of Latin America, respectively [2,3]. The vast majority of cases are reported in normal hosts and continuous exposure to infecting propagules in rural areas is considered to be the main risk factor for this condition [1].

PCM may present in two clinical forms: (i) an acute/subacute form, usually reported in children (or adults <30 years old), with high fever, disseminated lymphadenopathy, hepatosplenomegaly, skin lesions with limited or absent lung involvement and eventually bone lesions, among other symptoms; (ii) a chronic form that represents >80% of all cases reported, presenting with exuberant lung involvement, skin, mucocutaneous lesions or both, and eventually, lesions of the central nervous system and other organs [4,5]. In both clinical forms, adrenal involvement may take place. The polarization between clinical forms is related to the pattern of the adaptive T cell immune response, with a Th2 and Th9 response leading to an uncontrolled inflammatory process in the acute form, and a deficient Th1 mixed with a Th17 immune response leading to the chronic form of the disease [6,7]. Mortality rates of PCM in normal hosts is usually <5%, but sequelae are frequently documented and include chronic respiratory failure and Addison disease [5,8]. Diagnosis is mainly obtained by conventional methods, including direct examination, culture, histopathology, and detection of specific anti-*Paracoccidioides* antibodies (immunodiffusion or counterimmunoelectrophoresis). PCR-based methods and assays for specific antigen detection were developed by reference labs but are not available in the vast majority of the medical centers in Latin America [9].

Antifungal treatment of mild and moderate cases usually relies on itraconazole or the combination of sulfamethoxazole-trimethoprim. Severe and disseminated infections may require the use of amphotericin B formulations followed by consolidation therapy with itraconazole or sulfamethoxazole-trimethoprim. Patients are usually treated for 12–24 months, depending on clinical presentation [5].

All the aforementioned knowledge applies to PCM in the normal host, and data regarding PCM and immunocompromised patients are scarce and limited. Lack of its clinical suspicion, late diagnosis, and uncertainties about its management are factors that may negatively impact the outcomes of PCM in this population. The present paper describes the peculiarities in terms of natural history and challenges in the clinical management of PCM in patients with HIV, cancer, malignant hemopathies, solid organ transplantation, and immunobiological drugs. 

## 2. Material and Methods

### 2.1. Search Strategy

We searched the Pubmed database for reports of PCM in immunocompromised patients that were published in the last 30 years. We made all efforts to identify papers addressing epidemiology, fungal diagnosis and antifungal therapy in five different scenarios: HIV, cancer, hematologic patients, solid organ transplant, and related to use of immunobiological agents such as TNF inhibitors and anti-CD20 blockers. Search terms included various combinations of the terms “paracoccidioidomycosis” or “*Paracoccidioides*” with one of the following: “HIV”, “AIDS”, “cancer”, “leukemia”, “lymphoma”, “myelodysplastic syndrome”, “aplastic anemia”, “stem cell transplantation”, “kidney transplantation”, “liver transplantation”, “heart transplantation”, “lung transplantation”; “TNF inhibitor”; “infliximab”; “etanercept”; “adalimumab”; “anti-CD20”; “rituximab”; “natalizumab”. We reviewed all articles retrieved from these search terms and relevant references cited in those articles. There were no language restrictions and only cases with proven or probable PCM were included, following criteria defined elsewhere [5].

Data on age, sex, underlying diseases, immunological status, risk factors (e.g., rural work, tobacco or alcohol consumption), immunosuppressive therapy, laboratorial diagnosis, details of antifungal induction, maintenance therapies, as well as relapse and outcome were recorded. The outcome was considered to be favorable if the patient met the cure criteria according to the Brazilian Guidelines for the Clinical Management of PCM [5]. Recurrence of clinical signs and symptoms, or radiology findings in the presence of any laboratory results suggesting active PCM, were used to define a relapse episode. 

### 2.2. Statistical Analysis

Comparisons between groups were performed using Fisher’s exact or chi-square tests when appropriate for the categorical variables (SPSS v.25, IBM, Armonk, NY, USA). *p* values of <0.05 were considered to be statistically significant.

## 3. Results and Discussion

### 3.1. Paracoccidioidomycosis and HIV Infection

The first two cases of PCM associated with HIV infection were reported in 1989 [10]. Since then, PCM/HIV coinfection occurrence has been reported as small case-series in endemic areas in Brazil [11,12,13], and in isolated cases reports in Colombia and Argentina [14,15]. Two retrospective case-control studies have been conducted up until the present date. In the first study published in 2009, Morejón, Machado and Martinez reported 53 cases of PCM and HIV coinfection in Brazil [16]. In the second controlled study, Almeida et al. 2016, reported thirty-one HIV-infected patients with PCM between 1993 and 2014 [17]. After compiling the data from these two case-control studies [16,17], two case-series reports [13,18], and 30 single case reports [10,14,19,20,21,22,23,24,25,26,27,28,29,30,31,32,33,34,35], we retrieved 136 cases of PCM and HIV coinfection reported in the last 30 years. They aged between 13 and 59 years, with a mean age of 35.9 years. Twenty-six (19%) were female, and only two were in the adult PCM form (6%). The higher proportion of females in the casuistic of immunocompromised patients with PCM compared to the usual gender distribution observed in normal adult hosts (over 6%) suggests that the hormonal protection described for normal hosts is mitigated in the setting of immunosuppression [5]. Most patients worked in the urban area (68%), which is different from the usual epidemiology of the disease. Thus, an ancient exposure and activation of a latent infection might have occurred on these coinfected patients.

**Figure 1:**
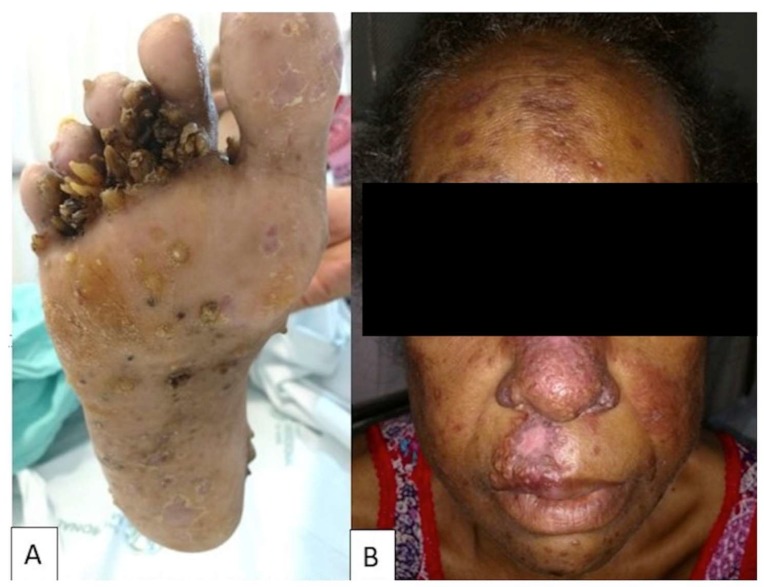
Skin involvement in PCM-HIV coinfection. (**A**) Verrucous lesions on the foot caused by hematogenous dissemination. (**B**) Papulonodular ulcerative lesions caused by hematogenous dissemination. Illustration provided by Prof. Paulo Mendes Peçanha from Infectious Disease Unit, Universidade Federal do Espírito Santo.

**Figure 2:**
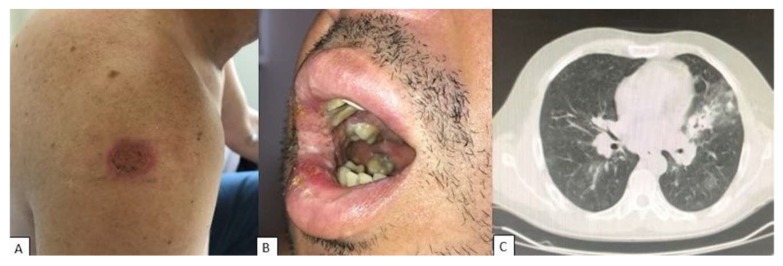
Clinical presentation of PCM-HIV coinfection. (**A**) Ulcerative lesions on the arm. (**B**) Moriform ulcerative perioral involvement. (**C**) Diffuse ground-glass opacities and consolidation in left superior lobe on chest computed tomography. Illustration provided by Dr. Adriana Maria Porro from the Dermatology Department, Escola Paulista de Medicina-Universidade Federal de São Paulo.

In 56 cases, data regarding the awareness of the HIV status at the PCM diagnosis were provided, and only 31 (55%) patients were known to be HIV-infected at that time. Over 80% of the PCM-HIV coinfected patients for whom CD4+ cell counts were available had <200 cells/mm^3^. This finding suggests that *Paracoccidioides* spp. may take advantage of the T-cell immunosuppression related to AIDS to shift from quiescent infection to systemic disease. Fever, generalized lymphadenopathy, splenomegaly, and skin lesions, which are generally reported in the acute form of the PCM disease, were more common in PCM-HIV coinfected patients than in the immunocompetent group (see Table 1, Figure 1 and Figure 2). 

Pulmonary disease was reported in most of the coinfected population despite the age of the patient, characterizing a mixed clinical form (juvenile–adult) exhibiting simultaneous lung involvement and disseminated infection of the reticuloendothelial system. This is a clinical presentation suggestive of an opportunistic manifestation of PCM in this specific population. This mixed clinical form, already reported by other authors, may be a consequence of the inefficient immune control of the primary lung foci followed by lymphohematogenous dissemination [36,37].

Coinfected patients presented more often with positive histopathology and culture results when compared to HIV-negative patients with PCM (see Table 1). These data reflect the higher fungal burden in the coinfected patients. In contrast, the detection of anti-*Paracoccidioides* serum antibody by quantitative double immunodiffusion test or counterimmunoelectrophoresis had lower positive rates in the PCM-HIV population (74.6% vs. 97.2%, *p* < 0.001). Therefore, in patients with AIDS, *Paracoccidioides* sp. negative serological results do not exclude the PCM diagnosis, which should be investigated with alternative microbiological tests and histopathological examination. 

Two case reports described PCM in patients receiving cotrimoxazole prophylaxis [24,38]. Similarly, Morejón et al. described 10 out 25 coinfected patients that were diagnosed with PCM under cotrimoxazole prophylaxis. Consequently, PCM should not be ruled out in patients under trimethoprim–sulfamethoxazole prophylaxis.

The overall mortality rate was higher in the coinfected population (35% vs. 7.9%, *p* < 0.001, see Table 1), mainly as a consequence of the severe immunodepression seen in most of the patients. Information about the presence of any other (non-PCM) concomitant opportunistic infections was mentioned by the authors in only 65 (47.7%) of 136 patients. Of note, only 19 cases (29%) of the 65 patients where this information was available had other severe infections concomitantly with PCM. This means that the outcome of PCM in this population was not impacted by other severe conditions in 71% of the cases. Fungemia by *Paracoccidioides* seems to be a predictor of poor prognosis since all three patients with positive blood cultures died. The choice of the initial antifungal therapy did not influence the outcome. Amphotericin B deoxycholate (AMB) was used as primary therapy in 19 cases, and 9 patients died (47.3%). Of note, all these nine patients with a fatal outcome had concomitant severe opportunist infections. Among 18 cases in which cotrimoxazole or itraconazole was used as initial therapy, seven died (38.8%, *p* = 0.7). Otherwise, one could suggest that there was one imbalance in the clinical severity of patients exposed to amphotericin B and other drugs that may certainly influence the expected mortality in both groups. 

### 3.2. Paracoccidioidomycosis and Solid Organ Malignancies

The relationship between PCM and solid cancer was first described more than 80 years ago [39]. Concomitant PCM and solid cancer are reported in 0.16 to 11% of the cases, according to some cohorts [39,40,41]. Most of the solid cancers are carcinomas (>80%) from the respiratory or digestive tract [42], and are related to the chronic form of PCM and its risk factors, such as male sex, rural workers with a history of smoking and alcohol intake [1,42]. Likewise, among the 36 cases that fulfilled the inclusion criteria (31 from two case series [42,43] and five from isolated case reports [44,45,46,47,48]), 26 (72%) were related to carcinomas of the upper and lower respiratory (*n* = 16, 44%) and digestive tracts, mainly the oropharynx and esophagus (*n* = 7, 19%). Carcinoma was diagnosed at the same anatomical site of the fungal lesion in twenty-one cases (58%). In thirteen cases (36%) PCM appeared before cancer, and in 19 patients (53%) cancer was diagnosed simultaneously with PCM. These findings have led researchers to raise the hypothesis that PCM may be an additive factor for cancer development due to chronic antigenic stimulation of the pathogenic yeasts on the epithelial cells [43].

**Figure 3:**
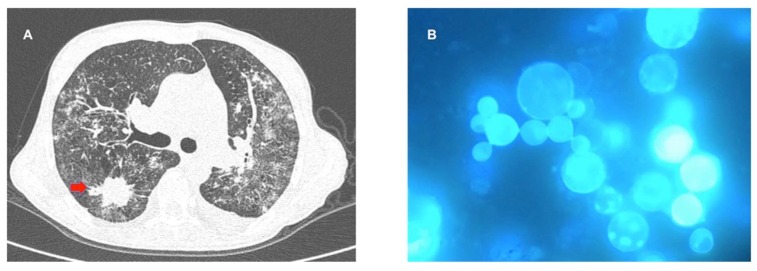
(**A**) Chest computed tomography scan showing spiculated nodule from adenocarcinoma (red arrow) and bilateral diffuse bronchoalveolar infiltrates from PCM. (**B**) Bronchoalveolar lavage with yeast cells with multiple daughter buds (Calcofluor white, 1000×). Figure **A** provided by Dr. Drielle Peixoto Bittencourt from Hospital do Cancer, Universidade de São Paulo; Figure **B** provided by the authors.

So far, we were not able to identify any particularity in terms of clinical presentation of PCM in patients with solid cancer. Serology was useful for PCM diagnosis in this population in only 50% of cases [43]. Figure 3 illustrates a case of concomitant pulmonary adenocarcinoma and PCM. In a retrospective analysis of 25 cases of PCM and cancer, Rodrigues et al. reported a mortality rate of 16%, a rate apparently higher than that associated to normal hosts [43,49]. 

### 3.3. Paracoccidioidomycosis and Hematologic Malignancies

Hematologic malignancies are rarely reported in patients with PCM, with an estimated prevalence of 0–3% [42]. A dozen cases were reported in the last 30 years, most of them were B cell lymphomas (either Hodgkin or non-Hodgkin) that were diagnosed after the PCM disease (1–8 years) [41,42,43,50]. Resende et al. reported four detailed cases of PCM and lymphoma, all of them had lymph nodes with PCM yeasts found in histopathology and positive serology [50]. The patients were treated for a long time, from 2 to 10 years, and two of them showed PCM recurrence. Two patients died due to complications related to the lymphoma [50]. The authors suggested that the chronic PCM antigenic stimulation may have had a role in the development of B cell lymphoma [50], but further investigation is necessary to confirm this hypothesis. The limited casuistic precludes any conclusion in terms of putative peculiarities of natural history or diagnostic tools for PCM in this specific setting.

### 3.4. Paracoccidioidomycosis and Solid Organ Transplant

**Figure 4:**
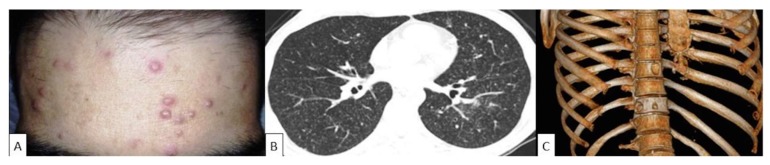
Disseminated paracoccidioidomycosis in a kidney transplant recipient. (**A**) Vesiculopapular lesions on the face. (**B**) Chest computerized tomography showing a miliary nodular pattern. (**C**) Chest computerized tomography and 3D reconstruction of the thoracic bone structure showing osteolytic vertebral lesions. Illustration provided by Dr. Daniel Wagner from Hospital do Rim, Universidade Federal de São Paulo.

Among the different solid organ transplant modalities, chronic PCM has been described predominantly in kidney transplant recipients. Nine cases of PCM in kidney transplant patients and a single episode in a liver transplant recipient fulfilled the inclusion criteria [51,52,53,54,55]. We excluded from the present series one episode of PCM mentioned in a report of a lung transplant recipient without any further details [56,57]. The patients had a median age of 55 years; three of them were rural workers before the transplantation, and three (60%) out of five cases with gender description were male. Seven cases developed PCM after one year of transplantation (range 1–14 years), none of them were having cotrimoxazole prophylaxis at diagnosis, and symptoms of PCM appeared 2 to 6 months before diagnosis. One case lacked a description of the time elapsed between transplant and PCM diagnosis [51]. Three (33%) of those reported cases had skin lesions, either combined with oral mucosal and lung infiltrates (two cases), or with lymphadenopathy (one case). One illustrative case is presented in Figure 4.

Three reports provided information regarding immunosuppressive therapy, that consisted of corticosteroids and the combination of other immunosuppressants, such as cyclosporine and azathioprine [52], mycophenolate mofetil (MMF) and tacrolimus [53], and mesalazine and tacrolimus [54]. The diagnosis was mainly achieved through direct examination or histopathology of clinical samples. All patients required hospitalization, four were initially treated with AMB formulations, three out nine patients (33%) died due to the mycosis. Of note, a 57-year-old female patient developed acute respiratory failure due to PCM only two days after the kidney transplantation [55]. This particular patient lived in a rural area and was diagnosed with a solitary pulmonary nodule before the transplant, which was considered to be latent tuberculosis. The pre-transplant immunosuppression therapy consisted of anti-thymocyte globulin (ATG) and MMF. The patient was successfully treated with liposomal AMB (1 mg/Kg) for 14 days followed by itraconazole (200mg/day) for one year. ID serology tested in the acute phase of the disease and during clinical follow-up were both negative. 

One case of severe disseminated PCM in a 3-year-old girl was reported 24 months after liver transplantation due to congenital biliary atresia [56]. She was initially treated with cotrimoxazole (200/40 mg, q12h), and due to a poor clinical response, AMB was also prescribed. Initial CIE serology was positive (titer 1/64), and after six months of cotrimoxazole (100/20 mg q12h) maintenance therapy, the patient was considered cured [56].

Prophylaxis with cotrimoxazole after transplantation explains the rarity of PCM in this scenario, as this drug is active against *P. brasiliensis*. Most of the reported cases occur after the first year of transplantation when immunosuppression is tapered and cotrimoxazole prophylaxis is no longer necessary. However, despite being rare, PCM in kidney transplant patients seems to have a poor prognosis, possibly related to its low clinical suspicion, difficult and late diagnosis (negative serological tests), and immunosuppression. Indeed, it has been demonstrated that immunosuppressed kidney transplant recipients have a persistent poor Th1 immune response to *Paracoccidioides* antigen gp43 [58].

Due to the rarity of PCM in organ transplant recipients, there is no formal recommendation for living donors and recipients that have lived in endemic areas to be routinely screened for PCM latent infection before transplant. Serological tests have poor value for the clinical management of PCM disease in kidney transplant recipients, and AMB lipid formulations have to be considered as initial therapy in severe cases [59].

### 3.5. Paracoccidioidomycosis and Immunotherapies

Only three cases of PCM disease related to immunobiological agents have been reported so far [60,61]. A 60-year-old man with rheumatoid arthritis and on immunosuppressive therapy, including methotrexate, leflunomide and adalimumab (40mg every 15 days), for 3 years, was hospitalized to investigate a chronic hip pain that was further diagnosed as an osteosarcoma. Chest CT-scan revealed an excavated pulmonary lesion on the lower left lobe and biopsy revealed granuloma and yeasts compatible with *P. brasiliensis*. Despite AMB induction therapy and adalimumab interruption, the patient developed sepsis after a hip surgery for tumor resection and died [60]. A 47-year old man with psoriatic spondyloarthritis and on infliximab therapy for 18 months developed fever and respiratory symptoms. CT-scan revealed a left inferior pulmonary lobe nodule and mediastinal lymphadenopathy. Lung biopsy histological analysis diagnosed PCM. Investigation of anti-*Paracoccidioides* antibodies by the immunodiffusion technique was negative. Infliximab was suspended and the patient was successfully treated with cotrimoxazole for 29 months. A 46-year old man with multiple sclerosis developed pulmonary PCM after 15 months of natalizumab therapy [62]. The diagnosis was made by lung biopsy and the patient was treated with itraconazole and natalizumab was discontinued.

In the USA, TNF inhibitors, mainly infliximab, have been related to histoplasmosis, with a median of 15 months after initiation of the immunobiological drug [63]. Likewise, the reported cases of PCM in patients undergoing TNF inhibitor therapy occurred more than one year after the introduction of the medication. These cases highlight the need to include PCM in the list of opportunistic infections in patients under long-term immunotherapy from endemic areas. Moreover, negative serology should not exclude the diagnosis of PCM in these patients and infection must be managed with interruption of the TNF blocker agent and prolonged antifungal therapy.

## 4. Conclusions

Despite being uncommon, PCM may be reported in patients with immunosuppression, including AIDS, cancer, patients with solid organ transplantations, or on immunobiological therapy. The vast majority of PCM in immunosuppressed patients has been reported in HIV patients, where this disease may exhibit simultaneously the clinical characteristics of chronic (lung involvement) and acute forms (generalized lymph adenomegaly and hepatosplenomegaly) of the disease. In this population, more disease relapses and higher mortality rates are reported than in non-immunocompromised hosts. 

Lymphoma was the most common hematologic malignancy reported with PCM, and few cases of PCM have been reported in the late period after kidney transplantation. We were not able to identify any change in terms of the natural history of PCM documented in patients with cancer or solid organ transplant recipients.

Diagnosis of PCM may be challenging in immunosuppressed patients where serology usually has a low sensitivity and PCR-based methods or assays for antigen detection are not available in the vast majority of routine laboratories. Consequently, invasive procedures (e.g., biopsy) may be required to confirm the diagnosis [17,62]. Of note, in endemic areas, paracoccidioidomycosis should be included in the differential diagnosis of any patient with a disease associated with T-cell immunodeficiency who presents with pulmonary infiltrates with nodules, cavitation or chronic alveolar consolidation, as well as skin or mucocutaneous lesions with a chronic evolution.

Regarding specific therapy of patients with severe clinical forms of PCM, amphotericin B should be promptly initiated, followed by 12 to 24 months of treatment with itraconazole or sulfamethoxazole-trimethoprim. Treatment duration relies on the severity of clinical presentation, sites of infection, restoration of the host immune response, as well as the clinical and laboratorial response to therapy [5].

Finally, immunocompromised patients who travel to endemic areas of PCM should be counseled before traveling to avoid high-risk exposures. Once the transplant recipient returns from an endemic area, the clinician must instigate a complete diagnostic investigation if any sign or symptom of PCM appears, in order to treat it rapidly and to mitigate against morbidity and a poor outcome [59].

## Figures and Tables

**Table 1:** Comparison of clinical, laboratory and outcome data from adult patients with paracoccidioidomycosis coinfected and non-coinfected with HIV virus.

	PCM and HIV (%)	PCM (%)	*p* Value
**Clinical Data**			
Fever	82.7	35.4 **	<0.001
Lymphadenopathy	72.9 ^#^	50.6 *	<0.001
Splenomegaly	22.6	4.7 *	<0.001
Skin Lesions	58.9	29.6 *	<0.001
Pneumopathy	70.3	63.8 *	0.15
Oral mucosa	29	50 *	<0.001
**Laboratory data (positivity rates)**			
Direct microscopy	57.4	44 **	0.052
Culture	42.2	25.3 *	<0.001
Histopathology	94.5	64.7 *	<0.001
Serology	74.6	97.2 *	<0.001
**Outcomes**			
Relapse rates	11	8.2 **	0.48
Mortality rates	35	7.9 **	<0.001

* Data extracted from the references: [2,4]; ** data extracted from the references [16,17]. ^#^ PCM coinfected patients usually present multiple lymph nodes involvement, in different anatomic sites.
